# m6A RNA modification in tumor-associated macrophages: emerging roles in cancer immunity

**DOI:** 10.3389/fimmu.2025.1693336

**Published:** 2025-10-02

**Authors:** Xi Chen, Shanrui Pu, Kun Lian, Lihua Li, Xiulin Jiang

**Affiliations:** ^1^ NHC Key Laboratory of Drug Addiction Medicine, Kunming Medical University, Kunming, China; ^2^ Key Laboratory of Neurological and Psychiatric Disease Research of Yunnan Province, The Second Affiliated Hospital of Kunming Medical University, Kunming, China; ^3^ School of Biosciences, University of Birmingham, Birmingham, United Kingdom; ^4^ Department of Systems Biology, City of Hope Comprehensive Cancer Center Biomedical Research Center, Monrovia, CA, United States

**Keywords:** m6A RNA modification, tumor-associated macrophages, tumor microenvironment, cancer immunotherapy, YTHDF2

## Abstract

N6-methyladenosine (m6A) is the most prevalent internal modification of eukaryotic mRNA and has emerged as a pivotal regulator of gene expression at the post-transcriptional level. In the tumor immune microenvironment, tumor-associated macrophages (TAMs) represent a highly plastic and heterogeneous population that profoundly influences cancer progression, immune evasion, and therapeutic response. Recent studies have uncovered that m6A modification, mediated by dynamic “writers,” “erasers,” and “readers,” exerts critical regulatory effects on TAM differentiation, polarization, and functional reprogramming. By modulating the stability, translation, and decay of transcripts involved in inflammatory signaling, metabolic adaptation, and immune checkpoints, m6A shapes the balance between tumor-promoting (M2-like) and tumor-suppressive (M1-like) macrophage phenotypes. Moreover, dysregulation of m6A machinery in TAMs has been linked to the suppression of anti-tumor immunity and resistance to immunotherapy, highlighting its translational potential as a therapeutic target. This review summarizes current advances in understanding the roles and mechanisms of m6A modification in TAM biology, discusses its implications in tumor immunity, and outlines the challenges and opportunities of targeting the m6A–TAM axis for cancer treatment.

## 1 Introduction

N6-methyladenosine (m6A) is the most prevalent internal modification in eukaryotic messenger RNAs (mRNAs) and long non-coding RNAs, dynamically regulated by methyltransferases (“writers”), demethylases (“erasers”), and m6A-binding proteins (“readers”) (1–3). Emerging evidence indicates that m6A modification influences nearly all aspects of RNA metabolism, including splicing, export, stability, and translation, thereby exerting profound effects on cellular fate and function (4, 5). Recent studies have highlighted the pivotal role of m6A in modulating immune cell development, activation, and effector functions, suggesting that RNA epigenetic modifications are integral to the regulation of immune responses (6–8).

Tumor-associated macrophages (TAMs) constitute a major component of the tumor microenvironment (TME) and are highly plastic, capable of adopting pro-inflammatory (M1-like) or immunosuppressive (M2-like) phenotypes depending on local cues (9, 10). TAMs contribute to tumor progression through multiple mechanisms, including promoting angiogenesis, suppressing cytotoxic T cell activity, remodeling the extracellular matrix, and secreting immunosuppressive cytokines. Despite the critical roles of TAMs in shaping anti-tumor immunity, the molecular mechanisms that regulate their functional plasticity remain incompletely understood (11–13).

Intriguingly, recent studies have begun to uncover a functional crosstalk between m6A RNA modification and macrophage biology (14, 15). m6A regulators can control macrophage polarization and inflammatory responses by modulating the stability and translation of key transcripts, such as cytokines, transcription factors, and signaling molecules (16). In the context of cancer, aberrant m6A modification in TAMs may contribute to their immunosuppressive phenotype, thereby promoting tumor immune evasion (17). This emerging evidence underscores the potential of m6A as a critical epigenetic layer linking RNA modification to the functional regulation of TAMs in the TME.

In this review, we summarize the current understanding of m6A RNA modification in TAMs, focusing on its roles in macrophage polarization, tumor-promoting functions, and interactions with other immune cells. We further discuss how m6A-mediated regulation of TAMs can influence anti-tumor immunity, providing insights into potential therapeutic strategies targeting RNA epigenetic modifications in cancer.

## 2 Overview of m6A regulatory machinery

**Figure 1:**
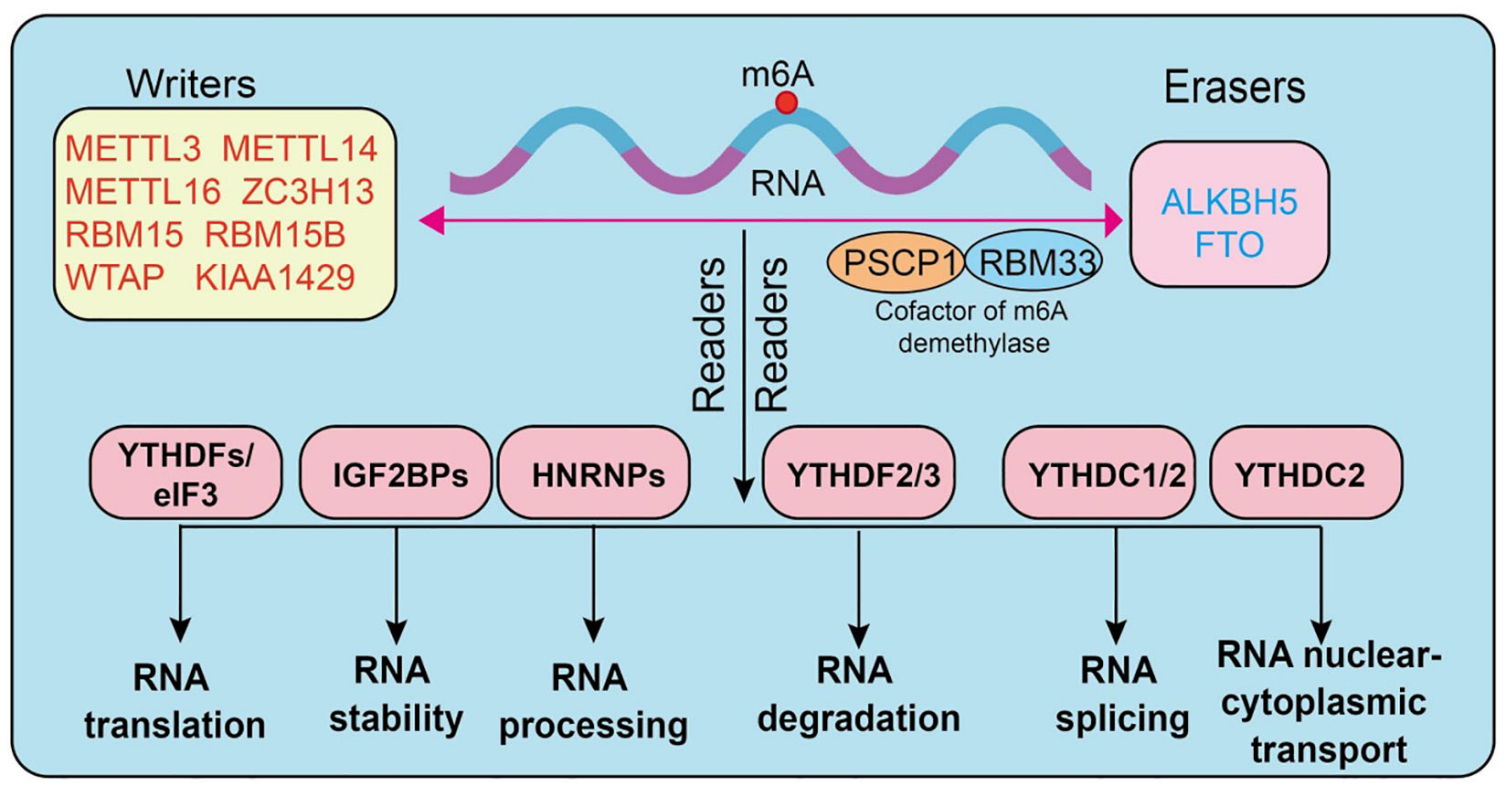
Dynamic process of m6A RNA modification and its core regulatory machinery.

N6-methyladenosine (m6A) is one of the most abundant endogenous chemical modifications in eukaryotic mRNA, playing a pivotal role in post-transcriptional gene regulation. Its dynamic and reversible nature relies on the coordinated actions of three major classes of regulatory proteins, termed “writers,” “erasers,” and “readers.” The methyltransferase complex constitutes the core “writers” of m6A modification. Among them, METTL3 serves as the primary catalytic subunit (18), METTL14 functions as an auxiliary subunit stabilizing the complex, and WTAP is responsible for substrate RNA localization and recruitment (7, 19–21). Additional regulators, including METTL5, METTL16, VIRMA (KIAA1429), RBM15/15B, ZC3H13, CBLL1, and ZCCHC4 (4, 22–27), contribute to controlling modification efficiency and site specificity. The “erasers” consist mainly of FTO and ALKBH5, two demethylases capable of efficiently removing m6A marks from RNA (28–31) (3), thereby ensuring the reversibility and dynamic equilibrium of this modification (32, 33). The “readers” are proteins that specifically recognize m6A sites and determine the fate of modified RNAs. The most classical readers are the YTH domain-containing family proteins (YTHDF1/2/3, YTHDC1/2) (34–36), which are functionally involved in translation promotion, RNA degradation, and splicing regulation (37, 38). The IGF2BP family (IGF2BP1/2/3) enhances the stability of target mRNAs by binding to m6A-modified sites. In addition, proteins such as HNRNPA2B1, HNRNPC, FMR1, EIF3A, ELAVL1, G3BP1, G3BP2, PRRC2A (39), and RBMX have also been shown to recognize or regulate m6A-marked RNAs, thus playing critical roles within the post-transcriptional regulatory network (7, 40–43) (**Figure 1**).

Importantly, emerging evidence indicates that these m6A regulators exert essential functions in immune cells, particularly in T cells, dendritic cells, and macrophages (44). For instance, METTL3-mediated m6A modification regulates T-cell differentiation, ALKBH5 modulates myeloid cell infiltration, and YTHDF family proteins are implicated in antigen presentation and inflammatory responses. These findings provide a theoretical foundation for understanding the potential roles of m6A in regulating TAM functions (45).

## 3 TAMs in the tumor microenvironment

Tumor-associated macrophages (TAMs) are among the most abundant immune cell populations within the tumor microenvironment (TME), originating from circulating monocytes or tissue-resident macrophages (46, 47). They display remarkable heterogeneity and plasticity under the influence of diverse tumor-derived and microenvironmental cues, exerting decisive roles in tumor immune regulation (48). Traditionally, TAMs have been classified into two extreme phenotypes: M1-like and M2-like. M1-like macrophages are activated by interferon-γ and Toll-like receptor (TLR) signaling, typically producing IL-12, TNF-α, and reactive oxygen species (ROS) (49–51). These macrophages are pro-inflammatory, enhance antigen presentation, and mediate antitumor activity. In contrast, M2-like macrophages are polarized in response to cytokines such as IL-4, IL-10, and IL-13. They are characterized by high expression of CD206, ARG1, and TGF-β, and are mainly involved in immunosuppression, tissue repair, and tumor promotion (52). Although the M1/M2 dichotomy provides a simplified framework for understanding macrophage biology, TAMs in actual tumors exist along a dynamic continuum of functional states. They may exhibit antitumor properties but are often reprogrammed by the TME toward protumor phenotypes (53, 54). Functionally, TAMs contribute to the establishment of an immunosuppressive TME through multiple mechanisms. They secrete inhibitory mediators such as IL-10, TGF-β, and PGE2, and directly suppress T-cell function through the expression of immune checkpoint molecules including PD-L1 and VISTA (55). Moreover, TAMs promote angiogenesis, extracellular matrix remodeling, and immune cell recruitment by releasing vascular endothelial growth factor (VEGF), matrix metalloproteinases (MMPs), and various chemokines, thereby facilitating tumor growth and metastasis (56, 57).

However, accumulating evidence indicates that TAMs in human tumors rarely conform strictly to the binary M1/M2 classification. Single-cell RNA sequencing and mass cytometry studies have revealed multiple TAM subtypes with distinct transcriptional programs and spatial localization (58). For instance, TAMs can be subdivided into inflammatory, angiogenic, lipid-associated, interferon-responsive, and tissue-resident-like populations, each contributing uniquely to tumor biology (59). In breast cancer, angiogenic TAMs expressing VEGF and MMP9 are concentrated near hypoxic tumor regions and support neovascularization (60). Interferon-responsive TAMs exhibit high expression of ISGs (interferon-stimulated genes) and may mediate anti-tumor immunity under certain conditions (61). Lipid-associated TAMs display enriched lipid metabolism gene signatures, contributing to metabolic remodeling in the TME and immunosuppression.

Furthermore, TAMs display dynamic plasticity, transitioning between subtypes in response to evolving tumor cues, chemokine gradients, and therapy-induced stress. This plasticity enables TAMs to simultaneously promote tumor progression, suppress adaptive immune responses, and regulate therapy resistance (62). Understanding the heterogeneity of TAM subtypes is therefore critical for designing strategies to reprogram TAMs toward tumor-inhibitory phenotypes. Notably, the molecular mechanisms underlying TAM heterogeneity involve epigenetic regulation, transcription factor networks, and post-transcriptional RNA modifications, including m6A methylation, which can influence polarization, cytokine production, and immunomodulatory functions.

TAMs also play critical roles in therapeutic responses. In chemotherapy, radiotherapy, and immune checkpoint blockade, TAMs frequently contribute to resistance by enhancing immunosuppression, promoting DNA repair, or remodeling the vascular microenvironment (63–65). Here, we provide a detailed summary of the differences between the two distinct types of tumor-associated macrophages, as shown in **Table 1**. Overall, TAMs possess dual properties: on one hand, they can participate in antitumor immunity through pro-inflammatory responses; on the other, they are more commonly reprogrammed by the TME into immunosuppressive and tumor-promoting states (66). Owing to this duality, TAMs have emerged as highly promising therapeutic targets in cancer immunotherapy (67). Strategies such as depletion, inhibition of recruitment, or functional reprogramming of TAMs hold great potential to enhance immunotherapeutic efficacy and improve patient outcomes.

**Table 1:** Comparative features of classically (M1) vs alternatively (M2) polarized macrophages.

Feature	M1 (classically activated)	M2 (alternatively activated/TAM-like)
Canonical polarizing cues	IFN-γ; LPS; TNF; GM-CSF; microbial products (TLR ligands)	IL-4/IL-13 (M2a); immune complexes + TLR/IL-1R signals (M2b); IL-10, TGF-β, glucocorticoids (M2c); tumor/TME factors—hypoxia, lactic acid, adenosine, PGE_2_, CSF-1, IL-6, IL-8, VEGF (M2-like TAMs)
Primary transcriptional programs	STAT1, IRF1/5, NF-κB, AP-1	STAT6, PPARγ/δ, KLF4, IRF4, c-Maf
Pattern-recognition & surface phenotype (human/mouse)	↑TLR2/4; ↑CD80, CD86, CD40; ↑MHC-II (HLA-DR/I-A/I-E); ↑CD64 (FcγRI); CCR7	↑CD206 (MRC1), CD163, CD204 (MSR1), MerTK; ↓co-stimulatory molecules; variable MHC-II; CX3CR1; CCR2low/CCR5/CCR1
Cytokines (dominant profile)	IL-12^high, IL-23, TNF, IL-1β, IL-6, type I IFNs	IL-10^high, TGF-β, IL-1Ra; low IL-12; amphiregulin
Chemokines produced	CXCL9, CXCL10, CXCL11 (Th1-attracting); CCL2, CCL3, CCL5 (context-dependent)	CCL17, CCL18* (human-enriched), CCL22, CCL24; CXCL12; pro-angiogenic CXCL8 (IL-8)
Arginine/NO pathway	iNOS (NOS2) → NO and peroxynitrite; citrulline/NO cycle	ARG1 (mouse-dominant), ornithine → polyamines and proline (matrix deposition); low NO
Redox & antimicrobials	High ROS/RNS; NADPH oxidase active; antimicrobial peptides ↑	Lower ROS/RNS; enhanced scavenging/efferocytosis; antioxidant programs (HO-1, Nrf2 targets)
Metabolic wiring	Aerobic glycolysis (Warburg-like); PPP ↑; truncated TCA (citrate, succinate accumulation); fatty-acid synthesis ↑; itaconate (IRG1) ↑	Oxidative phosphorylation ↑; fatty-acid oxidation ↑; intact TCA; mitochondrial biogenesis; glutamine metabolism supports UDP-GlcNAc (glycosylation)
Antigen presentation & T-cell priming	Strong APC: ↑MHC-II and co-stimulation; promotes Th1 and cytotoxic T-cell responses	Weaker APC; promotes Th2, Treg and exhaustion; expresses inhibitory ligands (e.g., PD-L1 variably)
Phagocytosis & clearance	Efficient pathogen phagocytosis and killing	High efferocytosis (apoptotic cell clearance); tissue-repair remodeling
Matrix/angiogenesis	Matrix-degrading but generally anti-angiogenic (via CXCL9/10/11); can secrete MMPs during inflammation	Pro-angiogenic (VEGF, PDGF, PlGF); MMP-2/9, cathepsins; ECM remodeling and fibrosis
Tumor-related activities	Tumoricidal potential; enhances antigen presentation and CTL/Th1 recruitment; inhibits tumor growth in some contexts	Pro-tumoral: supports immune suppression, invasion, angiogenesis, metastasis; fosters therapy resistance
Secreted mediators (selected)	TNF, IL-12, IL-1β, IL-6, type I IFN; chemokines CXCL9/10/11; HMGB1	IL-10, TGF-β, CCL17/18/22/24; VEGF, EGF, PDGF; galectins; MMPs; ARG1-driven metabolites
Exhaustion/immune-checkpoint landscape (TME)	Can express PD-L1/PD-L2 under strong IFN-γ/NF-κB but generally pro-inflammatory	Frequently high PD-L1/PD-L2, B7-H4, VISTA; secretes factors that induce T-cell dysfunction
Representative markers used in studies	CD80, CD86, CD40, HLA-DR (MHC-II), CD64, iNOS (NOS2), CCR7; cytokines IL-12/TNF	CD206 (MRC1), CD163, CD204 (MSR1), MerTK, ARG1* (mouse), YM1/Chil3* (mouse), Fizz1/Retnla* (mouse), PD-L1, VEGF

## 4 m6A modification in TAM Biology

As the most prevalent form of RNA epigenetic modification, N6-methyladenosine (m6A) has recently been demonstrated to play critical roles in the development, differentiation, and functional regulation of immune cells (68). In tumor-associated macrophages (TAMs), m6A modification modulates multiple signaling pathways and post-transcriptional regulatory mechanisms, thereby influencing their polarization, cellular metabolism, and immune effector functions, ultimately determining their functional states within the tumor microenvironment (69, 70). Growing evidence indicates that the m6A regulatory network is closely linked to the immunosuppressive properties of TAMs, providing new insights into the mechanisms underlying tumor immune evasion.

### 4.1 Functions and mechanisms of m6A methyltransferases in TAM

**Figure 2:**
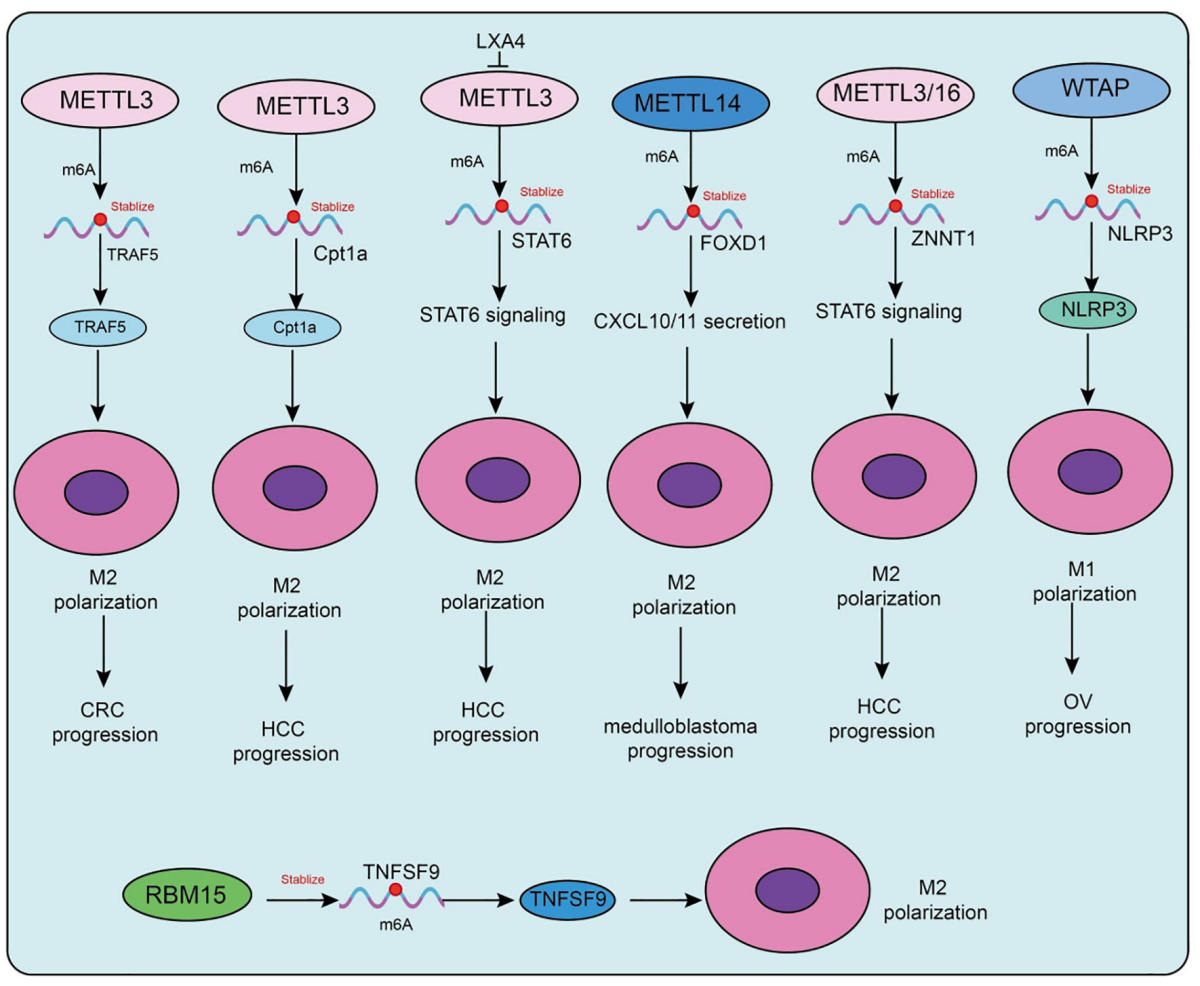
Functions and mechanisms of m6A “writers” (methyltransferases) in regulating tumor-associated macrophages.

The roles of m6A methyltransferases in tumor-associated macrophages (TAMs) are highly complex and context-dependent, with distinct tumor types and microenvironmental factors shaping their regulatory patterns (71) (**Table 2**; **Figure 2**). In colorectal cancer, studies have shown that M2-type TAMs promote oxaliplatin resistance through METTL3-mediated m6A modification, with TRAF5 identified as a critical downstream effector. This finding suggests that targeting the M2-TAM/METTL3 axis may represent a promising strategy to overcome chemoresistance (72). Conversely, in refractory thyroid carcinoma, M2-type TAMs transport miR-21-5p via extracellular vesicles to downregulate METTL3 expression in tumor cells, thereby reducing m6A modification of CD70 and stabilizing its expression (73). This process drives Treg and exhausted T-cell infiltration, ultimately leading to resistance to anti-PD-1 therapy (73). Notably, CD70 blockade effectively reverses this resistance. Further mechanistic investigations revealed that METTL3 collaborates with the m6A “reader” HNRNPA2B1 to regulate the m6A modification of pri-miR-146b, thereby promoting its maturation (74). Loss of miR-146b facilitates M2 polarization and activates PI3K/AKT signaling, enhancing immunosuppression and tumor progression. Moreover, deletion of METTL3 or miR-146b induces PD-L1 expression in TAMs via the p110β/PI3K/AKT pathway, thereby improving the efficacy of anti-PD-1 therapy (74).

**Table 2:** Functions and mechanisms of m6A methyltransferases in TAMs across cancers.

Cancer/disease context	Methyltransferase	Functional role in TAMs	Mechanism/downstream pathway	Consequence
Colorectal cancer (CRC)	METTL3	Promotes chemoresistance (oxaliplatin)	M2-TAMs upregulate METTL3 → m6A modification of TRAF5	Resistance to chemotherapy
Refractory thyroid carcinoma	METTL3 (in tumor cells, regulated by TAMs)	Promotes immune evasion, anti–PD-1 resistance	M2-TAMs transfer miR-21-5p via EVs → downregulate METTL3 in tumor cells → ↓m6A on CD70 → CD70 stabilized → Treg & exhausted T-cell infiltration	Resistance to anti–PD-1; reversed by CD70 blockade
Thyroid carcinoma (further mechanism)	METTL3 + HNRNPA2B1	Regulates pri-miR-146b maturation	Loss of miR-146b → M2 polarization + PI3K/AKT activation	Enhanced immunosuppression, PD-L1↑; loss improves anti–PD-1 efficacy
Hepatocellular carcinoma (HCC)	METTL3	Promotes M2 polarization & tumor progression	High-fat diet → ↑METTL3 stabilizes Cpt1a mRNA → fatty acid metabolism	Accelerated HCC progression; poor prognosis
Prostate cancer	METTL3	Promotes M2 polarization & tumor growth	Tumor-derived LXA4 suppresses METTL3 → STAT6 activation	M2 skewing; tumor progression (reversed by LXA4 receptor blockade)
Lung adenocarcinoma (LUAD)	METTL3	Promotes immune evasion & resistance	M2-TAMs ↑ METTL3 in LUAD cells → enhanced m6A modification	Tumor proliferation, invasion, migration, CTL resistance
Atherosclerosis	METTL3	Promotes pro-inflammatory M1 polarization	Stabilizes HDGF mRNA	Accelerates plaque formation

Metabolic factors are also involved in m6A-mediated regulation in TAMs. A high-fat diet upregulates METTL3 and Cpt1a expression in TAMs, promoting fatty acid metabolism and M2 polarization, which accelerates hepatocellular carcinoma (HCC) progression (75). Mechanistically, METTL3 stabilizes Cpt1a mRNA through m6A modification, driving metabolism-associated immunosuppression; deletion of Cpt1a effectively reverses this effect (75). Clinical samples further support this observation, showing that high METTL3 expression in TAMs correlates with increased M2 polarization, reduced M1 macrophages, and poor prognosis in HCC patients. In prostate cancer, tumor-derived lipid mediator LXA4 suppresses METTL3 expression in TAMs, activates STAT6 signaling, and promotes M2 polarization and tumor progression, while blockade of the LXA4 receptor reverses this effect (76). Similarly, in immunotherapy-resistant lung adenocarcinoma (LUAD), enrichment of M2-TAMs promotes tumor proliferation, invasion, migration, and resistance to CTL-mediated cytotoxicity. Mechanistically, M2-TAMs induce immune evasion in LUAD cells by upregulating METTL3 expression and m6A modification, whereas METTL3 interference reverses this phenotype (77).

Interestingly, in atherosclerosis, the function of m6A methyltransferases differs from that in tumors. Mettl3 promotes M1 polarization and inflammatory responses by stabilizing HDGF expression, thereby accelerating plaque formation, indicating a pro-inflammatory rather than protumorigenic role in this context (78). In addition to METTL3, METTL14 also plays pivotal roles. In SHH-type medulloblastoma (SHH-MB), TAM-derived exosomes transfer specific microRNAs that downregulate METTL14 expression in tumor cells, leading to reduced global m6A modification (79). FOXD1 has been identified as a key downstream target, whose high expression correlates with poor prognosis. FOXD1 silencing enhances CXCL10/11 secretion and CD8^+^ T-cell infiltration, significantly improving antitumor immunity when combined with PD-1 blockade *in vivo*. Similarly, in colorectal cancer (79), METTL14 suppresses EBI3 expression in TAMs, thereby limiting CD8^+^ T-cell dysfunction (80). Loss of METTL14, however, induces C1q^+^ TAM-mediated EBI3 secretion, which promotes CD8^+^ T-cell exhaustion and tumor progression (80). In cervical cancer, METTL14 enhances glycolysis-driven lactate production, which induces PD-1 overexpression in TAMs, impairing their phagocytic activity and reinforcing immunosuppression. Conversely, in HCC, M1-type TAMs transport miR-628-5p via exosomes to suppress METTL14 expression, thereby blocking m6A modification and nuclear export of circFUT8, ultimately exerting antitumor effects (81). A more complex feedback loop has been uncovered in HCC, in which METTL3/METTL16-mediated m6A modification stabilizes and upregulates ZNNT1, promoting OPN secretion that recruits and polarizes TAMs toward an M2 phenotype. In turn, S100A9 secreted by M2-TAMs enhances ZNNT1 expression through the AGER/NF-κB pathway, forming a ZNNT1/OPN/S100A9 positive feedback loop that accelerates malignant progression (82). Beyond METTL3, METTL14, and METTL16, WTAP also contributes to immune regulation. In a corneal alkali burn model, WTAP promotes both angiogenesis and lymphangiogenesis through the SUV39H1/CCL2 axis and the HIF-1α/VEGFA/C/D pathway, thereby accelerating corneal neovascularization (83). In ovarian cancer, TAM-derived CXCL16 activates the CXCL16–CXCR6 signaling pathway, resulting in downregulation of WTAP and upregulation of ALKBH5 in tumor cells, which alters m6A modification and enhances cisplatin resistance (84). Conversely, in atherosclerosis, WTAP stabilizes NLRP3 mRNA to promote pyroptosis and M1 polarization, accelerating disease progression, whereas WTAP knockdown significantly alleviates lesions, indicating its potential as a therapeutic target (85). In breast cancer, RBM15 promotes M2 polarization and paclitaxel resistance by upregulating TNFSF9 expression via m6A modification (86). Inhibition of RBM15 or TNFSF9 reverses M2 polarization and restores chemosensitivity. Lastly, in sepsis-associated acute lung injury (SA-ALI), ZC3H13 aggravates ferroptosis by promoting m6A modification and YTHDF2-dependent degradation of PRDX6 mRNA, thereby reducing PRDX6 levels. Overexpression of PRDX6 or silencing ZC3H13 alleviates lung injury, whereas combined loss abrogates the protective effects (87).

Collectively, m6A methyltransferases in TAMs participate in immune microenvironment remodeling through diverse mechanisms, including regulation of cellular metabolism, cytokine secretion, immune checkpoint expression, and noncoding RNA modification. Their roles are bidirectional: they may promote immunosuppression and tumor progression, but under certain conditions they can enhance antitumor or pro-inflammatory responses. Thus, selective modulation of m6A methyltransferases, tailored to tumor type and immune microenvironmental context, holds significant promise as a future strategy for precision immunotherapy.

### 4.2 Functions and mechanisms of m6A Demethylases in TAM

**Figure 3:**
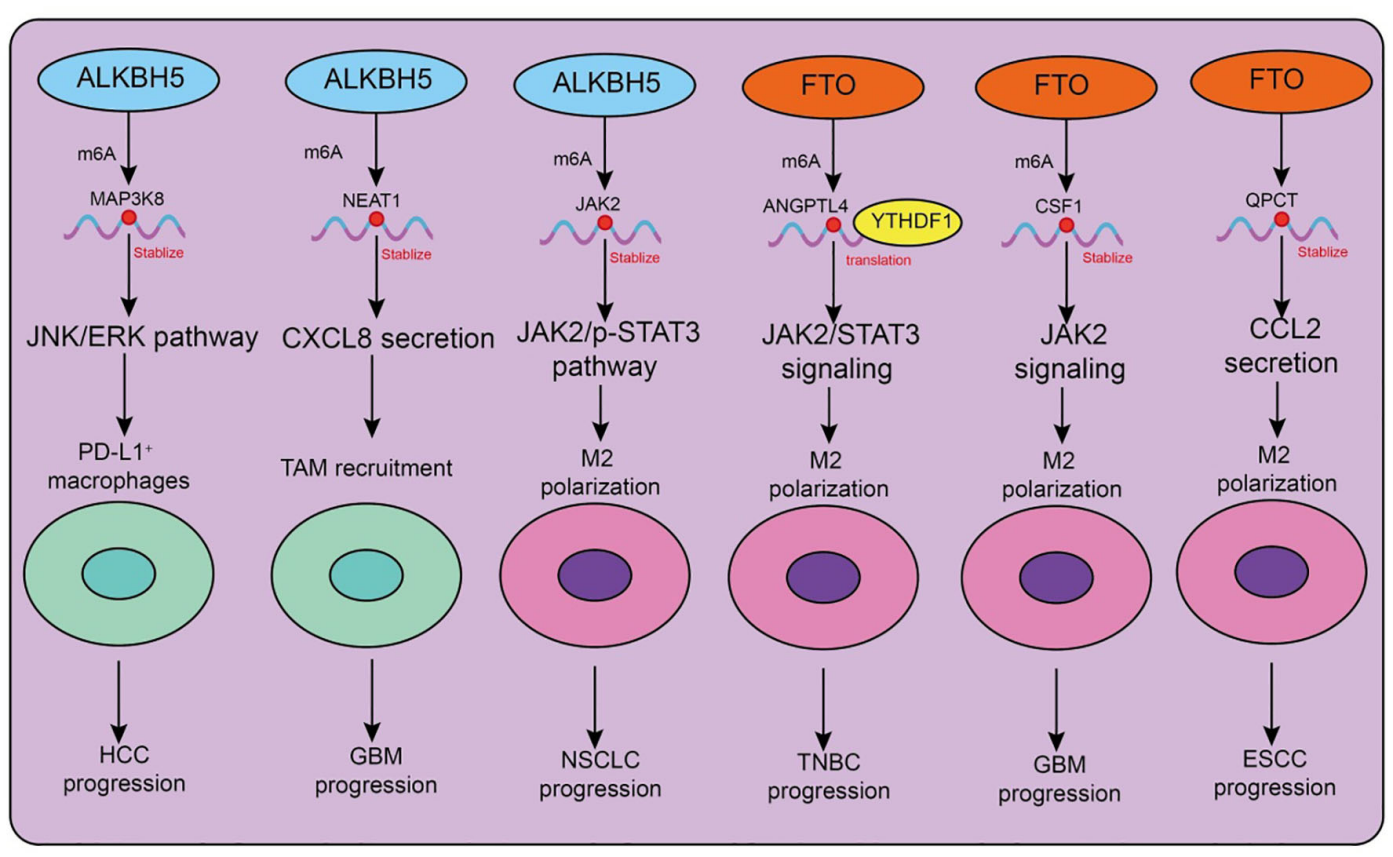
Functions and mechanisms of m6A “erasers” (demethylases) in regulating tumor-associated macrophages.

The major m6A demethylases, ALKBH5 and FTO, play critical roles in regulating the recruitment, polarization, and immune functions of tumor-associated macrophages (TAMs) (**Table 3**, **Figure 3**). Depending on tumor type and microenvironmental context, these enzymes modulate TAM activity through diverse signaling pathways, thereby influencing tumor progression and therapeutic responses. In hepatocellular carcinoma (HCC), ALKBH5 is highly expressed and closely associated with poor prognosis. Mechanistically, ALKBH5 promotes tumor cell proliferation and metastasis through m6A-dependent upregulation of MAP3K8, which activates the JNK/ERK pathway, while simultaneously inducing IL-8 expression to drive the recruitment of PD-L1^+^ macrophages. This process contributes to the establishment of an immunosuppressive microenvironment, highlighting the ALKBH5/MAP3K8 axis as a potential therapeutic target (88). Similarly, in glioblastoma (GBM), hypoxic conditions induce ALKBH5 upregulation, which removes m6A modifications from the long noncoding RNA NEAT1, stabilizing its transcript and facilitating paraspeckle formation. This, in turn, relieves transcriptional repression of CXCL8, enhances its secretion, promotes TAM recruitment, and accelerates the formation of an immunosuppressive milieu that drives tumor progression (89). In non-small cell lung cancer (NSCLC), ALKBH5 exhibits particularly complex roles. On the one hand, ALKBH5 expression correlates with PD-L1 levels, macrophage infiltration, and response to immunotherapy. Mechanistically, it stabilizes JAK2 mRNA to activate the JAK2/p-STAT3 pathway, while inducing the secretion of CCL2 and CXCL10 to recruit PD-L1^+^ TAMs and drive M2 polarization (90). Additionally, ALKBH5 cooperates with TAM-derived IL-6 to further enhance immunosuppression. On the other hand, smoking induces M2-TAM–derived extracellular vesicles (EVs) carrying circEML4, which are transferred into NSCLC cells and impair the nuclear localization of ALKBH5 (91). This leads to elevated m6A modification and malignant progression through the SOCS2–JAK/STAT pathway. Notably, circEML4^+^ M2-TAMs are significantly enriched in smoking patients, suggesting diagnostic potential (91). Further studies have revealed that CDCA4 m6A modification in NSCLC cells is co-regulated by METTL3 and ALKBH5. Through YTHDC2-mediated mechanisms, CDCA4 promotes cell proliferation and migration while altering the M1/M2 ratio, thereby reshaping the tumor immune microenvironment (92). Thus, the METTL3/ALKBH5–CDCA4–YTHDC2 axis represents a key regulatory pathway in NSCLC progression (92).

**Table 3:** Functions and mechanisms of m6A demethylases in TAMs across cancers and diseases.

Cancer/disease context	Demethylase	Functional role in TAMs	Mechanism/downstream pathway	Consequence
Hepatocellular carcinoma (HCC)	ALKBH5	Promotes immunosuppression & tumor progression	Upregulates MAP3K8 (JNK/ERK activation) + induces IL-8 secretion → recruits PD-L1^+^ TAMs	Poor prognosis; immunosuppressive TME
Glioblastoma (GBM)	ALKBH5	Drives TAM recruitment under hypoxia	Removes m6A from NEAT1 → stabilized lncRNA → paraspeckle formation → CXCL8 secretion ↑	TAM infiltration; immunosuppression; tumor progression
Non-small cell lung cancer (NSCLC)	ALKBH5	Promotes TAM recruitment & M2 polarization	Stabilizes JAK2 mRNA → JAK2/p-STAT3 activation; induces CCL2/CXCL10 secretion; cooperates with TAM-derived IL-6	PD-L1^+^ TAM infiltration; immune evasion
NSCLC (smoking-related)	ALKBH5 (suppressed by TAM EVs)	Facilitates tumor progression	M2-TAM EVs deliver circEML4 → blocks ALKBH5 nuclear localization → ↑m6A → activates SOCS2–JAK/STAT pathway	Malignant progression; enriched in smokers
NSCLC (further axis)	ALKBH5 + METTL3	Alters macrophage polarization	Co-regulate CDCA4 m6A modification → YTHDC2-dependent stabilization → proliferation/migration & altered M1/M2 ratio	Reshaped immune microenvironment; tumor progression
Triple-negative breast cancer (TNBC)	FTO (suppressed by D-2HG)	Induces M2 polarization & metastasis	D-2HG ↓FTO → ↑m6A → YTHDF1-mediated translation of ANGPTL4 → activates integrin–JAK2/STAT3	Tumor proliferation, metastasis, M2 TAM polarization
Gastric cancer	FTO	Promotes M2 polarization & tumor development	CAFs use FTO to upregulate NNMT	Enhanced immunosuppressive TME; tumor progression
Glioblastoma (GBM)	FTO	Promotes M2 polarization & immune remodeling	Fusion cells (tumor + MSCs) ↑ FTO → demethylates CSF1 mRNA → CSF1 secretion ↑	TAM M2 polarization; immune suppression
Esophageal squamous cell carcinoma (ESCC)	FTO (suppressed by FOXF2–RNF144A)	Restrains M2 polarization (antitumor effect)	FOXF2 induces RNF144A-mediated FTO degradation → ↓m6A demethylation	Reduced M2 TAMs; antitumor immunity
Lung adenocarcinoma (LUAD)	FTO	Promotes TAM recruitment & M2 polarization	Stabilizes QPCT mRNA → ↑CCL2 secretion	Immunosuppression; tumor progression
Silicosis (non-malignant)	FTO	Regulates macrophage polarization in fibrosis	FTO downregulation → M1 polarization & glycolysis ↑ (protective); FTO upregulation → M2 polarization	FTO↑ worsens fibrosis; FTO↓ alleviates fibrosis

Beyond ALKBH5, FTO also plays important roles in TAM regulation. In triple-negative breast cancer (TNBC), the metabolite D-2HG suppresses FTO activity, enhancing m6A modification and promoting YTHDF1-dependent translation of ANGPTL4 mRNA. ANGPTL4 activates integrin–JAK2/STAT3 signaling in an autocrine manner to promote tumor proliferation and metastasis, while also exerting paracrine effects to induce TAM M2 polarization, thereby accelerating tumor progression (93). Similarly, in gastric cancer, cancer-associated fibroblasts (CAFs) rely on FTO-mediated demethylation to upregulate NNMT, which promotes M2 polarization and tumor development (94). In GBM, hybrid cells formed by the fusion of tumor cells and mesenchymal stem cells employ FTO to demethylate CSF1 mRNA, enhancing its secretion and driving TAM M2 polarization, ultimately reshaping the immune landscape (95). Interestingly, FTO may exert opposing effects in different tumor and disease contexts. In esophageal squamous cell carcinoma (ESCC), the transcription factor FOXF2 promotes RNF144A-mediated ubiquitination and degradation of FTO, reducing m6A demethylation, inhibiting M2 polarization, and exerting antitumor effects. This identifies the FOXF2–RNF144A–FTO axis as a potential therapeutic target (96). In contrast, in lung adenocarcinoma (LUAD), FTO stabilizes QPCT mRNA and enhances CCL2 secretion, promoting TAM recruitment, M2 polarization, and immunosuppression, thereby accelerating tumor progression. Beyond cancer, FTO expression also influences macrophage polarization in non-malignant diseases such as silicosis: FTO downregulation promotes M1 polarization, enhances glycolysis, and alleviates pulmonary fibrosis, whereas FTO upregulation drives M2 polarization and exacerbates fibrotic pathology (97).

Collectively, ALKBH5 and FTO profoundly influence TAM recruitment, polarization, and immune function across diverse tumor and disease settings. Both enzymes can promote immunosuppression and malignant progression, yet under certain conditions, they may also inhibit M2 polarization and exert antitumor effects. Thus, the functions of m6A demethylases in TAMs are highly context-dependent, and their associated signaling axes-such as ALKBH5/MAP3K8, FTO/ANGPTL4, and FOXF2–RNF144A-FTO-represent promising therapeutic intervention targets.

### 4.3 Functions and mechanisms of m6A readers in tumor-associated macrophages

**Figure 4:**
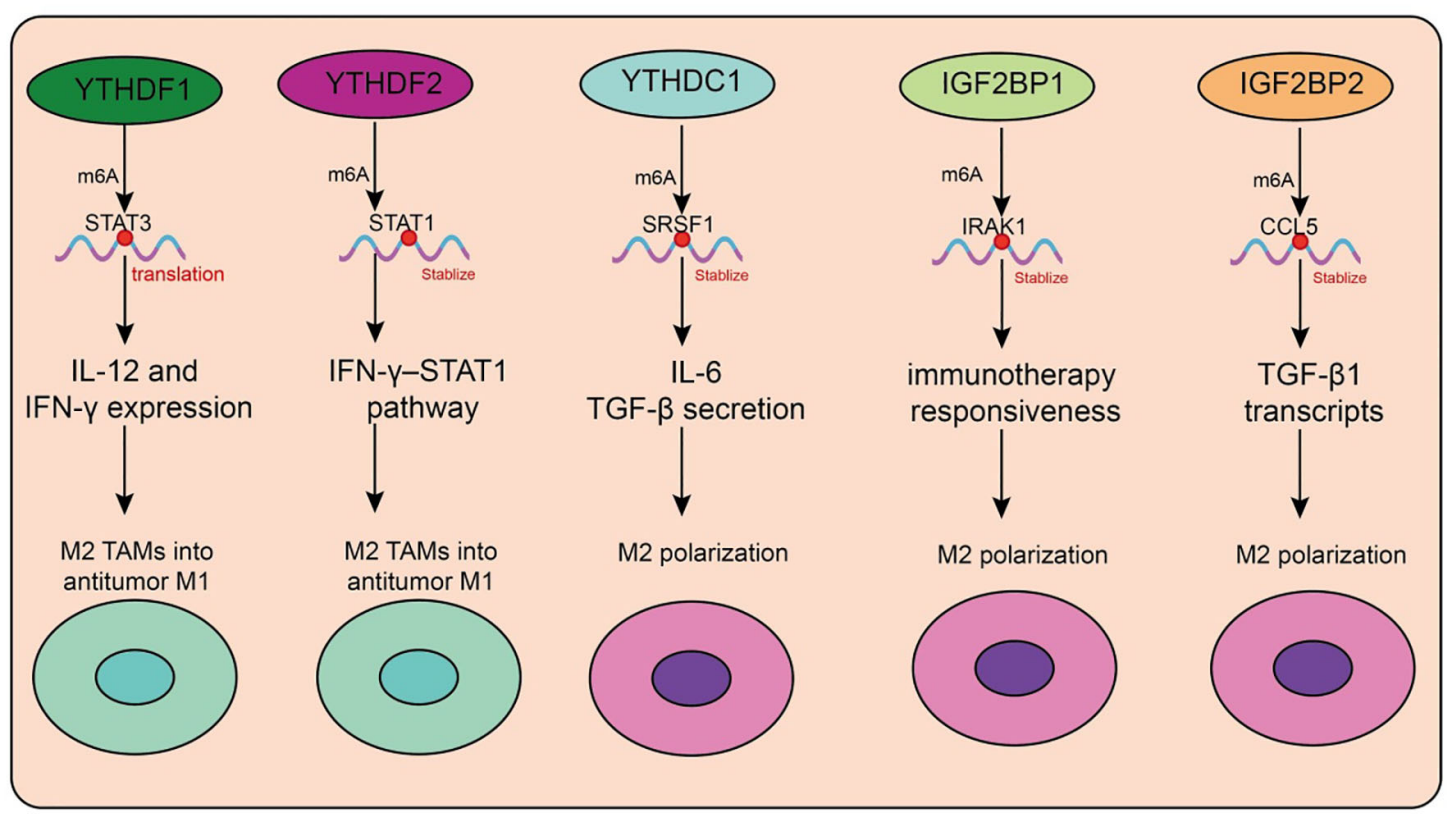
Functions and mechanisms of m6A “readers” (binding proteins) in regulating tumor-associated macrophages.

In recent years, the role of m6A reader proteins in the tumor immune microenvironment (TME) has attracted increasing attention, particularly regarding its regulation of tumor-associated macrophage (TAM) polarization and function (**Table 4**, **Figure 4**). Targeting the m6A reader YTHDF1 has shown promising therapeutic potential. A recent study developed a photosensitive dual-targeting nanoparticle system (M.RGD@Cr-CTS-siYTHDF1 NPs), which selectively delivers YTHDF1 siRNA into TAMs by simultaneously targeting integrin receptors on tumor cells and CD206 receptors on TAMs, in combination with photothermal activation. Mechanistically, silencing YTHDF1 in TAMs disrupts the STAT3–STAT1 balance (decreasing STAT3 while enhancing STAT1), thereby reprogramming immunosuppressive M2 TAMs into antitumor M1 phenotypes (98). This transition is accompanied by reduced IL-10 and increased IL-12 and IFN-γ expression, enhanced CD8^+^ T-cell infiltration, and diminished Treg accumulation (98). Functionally, the combined nanoparticle and photothermal strategy not only directly eradicates tumor cells but also promotes immune activation within the TME, highlighting its potential as a novel molecularly targeted immunotherapy platform. Similarly, YTHDF2 plays a pivotal role in TAM function. YTHDF2 promotes immunosuppressive polarization, whereas its deficiency activates the IFN-γ–STAT1 pathway, reprograms TAMs into an antitumor state, and enhances antigen cross-presentation to CD8^+^ T cells, thereby suppressing tumor progression (99). Further studies demonstrated that YTHDF2 expression in TAMs is driven by IL-10–STAT3 signaling. Therapeutically, conjugating siRNA against YTHDF2 with TLR9 agonists not only remodels TAM function and inhibits tumor growth but also enhances the efficacy of anti–PD-L1 therapy (99). In triple-negative breast cancer (TNBC), YTHDF2 reinforces tumor-promoting polarization and impairs antigen presentation, thereby contributing to chemoresistance. Knockdown of YTHDF2 enhances antitumor activity, and single-cell analyses revealed that transcription factors such as SPI1 are upregulated in YTHDF2-high macrophages, suggesting that YTHDF2 and its downstream effectors represent potential targets for overcoming TNBC chemoresistance (100). Beyond the YTHDF family, m6A-dependent regulation involving long noncoding RNAs (lncRNAs) also shapes TAM function. In pancreatic cancer, TAMs highly express lncRNA H19, which correlates with advanced stage and poor prognosis. H19 promotes M2 polarization and enhances IL-6, IL-10, and TGF-β secretion, thereby accelerating tumor progression (101). Mechanistically, H19 competitively binds miR-107 to modulate YTHDC1 expression and interacts directly with YTHDC1 protein, thereby regulating SRSF1 stability and alternative splicing of immunosuppressive cytokines (101). Organoid and PDX models suggest that ruxolitinib may represent a potential therapeutic option for patients with high H19 expression. In hepatocellular carcinoma (HCC), another study revealed that H3K36me3-guided m6A modification stabilizes lncRNA L1CAM-AS1 through IGF2BP1 recognition. L1CAM-AS1 prevents RAN ubiquitination by blocking its interaction with the E3 ligase OSTM1, thereby stabilizing RAN protein, activating NF-κB signaling, and upregulating CCL2 to recruit M2 TAMs (102). Reciprocal reinforcement occurs as M2 TAMs secrete CCL5, further activating NF-κB in HCC cells, establishing an immunosuppressive feedback loop. Targeting the L1CAM-AS1–RAN–NF-κB axis effectively reprograms TAMs and enhances PD-1 blockade efficacy (102).

**Table 4:** Roles of m6A-related regulators in tumor-associated macrophage (TAM) polarization and cancer immunity.

Regulator/molecule	Cancer context	Mechanism in TAMs	Immune consequence	Therapeutic implication
YTHDF1	General tumors	Silencing disrupts STAT3–STAT1 balance → reprograms M2 → M1; ↓ IL-10, ↑ IL-12/IFN-γ; ↑ CD8^+^ T-cell infiltration, ↓ Tregs	Promotes antitumor immunity	Dual-targeting nanoparticles (M.RGD@Cr-CTS-siYTHDF1 NPs) + photothermal therapy
YTHDF2	Multiple tumors, TNBC	Promotes immunosuppressive M2 polarization; deficiency activates IFN-γ–STAT1 pathway and enhances antigen cross-presentation	Antitumor reprogramming upon knockdown; ↓ chemoresistance in TNBC	siYTHDF2 + TLR9 agonists enhance anti–PD-L1 therapy
lncRNA H19	Pancreatic cancer	Promotes M2 polarization via miR-107/YTHDC1 axis; stabilizes SRSF1 → ↑ IL-6, IL-10, TGF-β	Immunosuppressive cytokine secretion	Ruxolitinib as potential option for high H19 tumors
lncRNA L1CAM-AS1	HCC	Stabilized by IGF2BP1 via H3K36me3-guided m6A; prevents RAN ubiquitination → activates NF-κB → ↑ CCL2, recruits M2 TAMs	Establishes immunosuppressive TAM feedback loop (CCL2/CCL5)	Targeting L1CAM-AS1–RAN–NF-κB axis enhances PD-1 blockade
circ_0020095	Colorectal cancer	Tumor exosomal circRNA taken up by TAMs; competes with IGF2BP1 → ↓ IRAK1 mRNA stabilization → blocks M1 polarization	Drives M2 phenotype, immunosuppression	Potential biomarker & IGF2BP1 modulation
IGF2BP2	Breast cancer	Enriched in immune-escape epithelial subsets; promotes CCL2-mediated M2/SPP1^+^ TAM recruitment	Enhances angiogenesis and immunosuppression	Marker of poor prognosis; targetable with immunotherapy
circNEIL3 → IGF2BP3	Glioma	Exosomal circNEIL3 stabilizes IGF2BP3 in TAMs → immunosuppressive polarization	Promotes tumor progression	IGF2BP3 inhibition synergizes with immunotherapy
IGF2BP3	HCC	Stabilizes CCL5 & TGF-β1 mRNAs → ↑ M2 TAM infiltration, ↓ CD8^+^ T cells	Suppresses antitumor immunity	Dual blockade of IGF2BP3 + CD47 enhances immune response
Exosomal miR-184-3p	Multiple tumors	Packaged via hnRNPA2B1; in TAMs → suppresses JNK, targets EGR1 → induces M2 polarization	Promotes immunosuppressive TAMs	Blocking hnRNPA2B1 or miR-184-3p release suppresses tumor growth

Circular RNAs (circRNAs) also modulate TAM function via m6A readers. In colorectal cancer, tumor-derived exosomal circ_0020095 is taken up by TAMs, suppressing M1 polarization and enhancing M2 phenotypes (103). Mechanistically, circ_0020095 competes with IGF2BP1 for binding, thereby impairing IRAK1 mRNA stabilization and blocking M1 polarization. Similarly, in breast cancer, unsupervised clustering analyses identified IGF2BP2 as a key factor associated with poor prognosis and reduced immunotherapy responsiveness (103). Single-cell data showed IGF2BP2 enrichment in immune-escape epithelial subsets, where it promotes CCL2-mediated macrophage recruitment, fostering M2-like and SPP1^+^ TAMs that drive angiogenesis and immunosuppression. In glioma, circNEIL3, generated through EWSR1-mediated circularization, stabilizes IGF2BP3 by preventing its ubiquitination, thereby promoting tumor progression. Tumor-derived exosomal circNEIL3 is transferred into TAMs, where it similarly stabilizes IGF2BP3, driving immunosuppressive polarization and accelerating glioma growth (104). In HCC, IGF2BP3 stabilizes CCL5 and TGF-β1 transcripts, facilitating TAM infiltration and M2 polarization, while impairing CD8^+^ T-cell activation. Dual blockade of IGF2BP3 and CD47 enhances antitumor immunity in preclinical models (105). Exosomal microRNAs also represent critical mediators in TAM regulation. Tumor cells selectively package miR-184-3p into exosomes via hnRNPA2B1, releasing it into the TME (106). In tumor cells, miR-184-3p promotes proliferation and metastasis by derepressing MAML1, while in TAMs it suppresses the JNK pathway and targets EGR1 to induce M2 polarization (106). Nanoparticle delivery of the c-Myc inhibitor JQ1 reduces Rac1-dependent exosome uptake, thereby blocking TAM reprogramming. Inhibiting hnRNPA2B1 or preventing exosomal miR-184-3p release significantly suppresses tumor growth and metastasis (106).

Collectively, these studies reveal the multilayered roles of m6A readers—including YTH family proteins, IGF2BP proteins, lncRNAs, circRNAs, and exosomal microRNAs-in orchestrating TAM polarization, cytokine signaling, and intercellular communication. By driving immunosuppressive TMEs, they contribute critically to tumor progression and therapeutic resistance. Deciphering these mechanisms not only deepens our understanding of m6A-mediated immune regulation but also provides promising strategies for the development of next-generation immunotherapies.

### 4.4 Relationship between m6A and TAM subtypes

Tumor-associated macrophages (TAMs) exhibit remarkable plasticity within the tumor immune microenvironment, with a spectrum of phenotypes ranging from pro-inflammatory, anti-tumorigenic M1-like macrophages to immunosuppressive, pro-tumorigenic M2-like macrophages (10). Emerging evidence suggests that N6-methyladenosine (m6A) RNA modification and its regulatory proteins (writers, erasers, and readers) play pivotal roles in shaping TAM polarization. On one hand, the methyltransferases METTL3 and METTL14 have been shown to stabilize transcripts of transcription factors and signaling molecules such as STAT1 and IRF5 through m6A modification, thereby promoting M1 polarization and enhancing anti-tumor immune responses (72). In contrast, the demethylases FTO and ALKBH5 are frequently associated with M2-like polarization, as they remove m6A marks to stabilize mRNAs encoding M2-related regulators including STAT6, PPARγ, and IL-10, thus supporting the immunosuppressive phenotype (90). In addition, m6A reader proteins contribute to the fine-tuning of TAM subsets. For example, YTHDF2 promotes the degradation of inflammatory transcripts, which may dampen M1 functions, whereas YTHDF1 enhances antigen presentation and translation efficiency, thereby potentially strengthening M1 activity under certain conditions (99). Collectively, these findings highlight the dynamic regulatory network of m6A modification in TAM polarization: METTL3/METTL14 tend to favor M1 differentiation, FTO/ALKBH5 facilitate M2 polarization, and reader proteins act as modulators to balance the two states. Such insights not only underscore the importance of m6A in dictating TAM plasticity, but also provide a rationale for targeting specific m6A regulators to reprogram TAMs and potentiate anti-tumor immunity.

## 5 Therapeutic potential and challenges of targeting m6A regulators to reprogram TAMs

Translating basic research on m6A RNA modification into therapeutic strategies represents a promising avenue for cancer treatment. Targeting m6A regulators in tumor-associated macrophages (TAMs) may reshape the immunosuppressive tumor microenvironment and enhance anti-tumor immunity. Therapeutic approaches can be discussed according to different classes of m6A regulators:

### 5.1 Targeting m6A “writers” (methyltransferases)

Methyltransferases such as METTL3 and METTL14 catalyze m6A installation on RNA transcripts, regulating macrophage polarization, cytokine production, and tumor-promoting functions. Inhibition of METTL3 has been shown to suppress the immunosuppressive phenotype of TAMs, reduce pro-tumor cytokines, and restore cytotoxic T cell activity in preclinical models (76). Small-molecule inhibitors or RNA interference targeting METTL3/METTL14 in TAMs may therefore represent a strategy to reprogram the TME toward anti-tumor immunity.

### 5.2 Targeting m6A “erasers” (demethylases)

Demethylases such as FTO and ALKBH5 remove m6A marks, thereby stabilizing or destabilizing key transcripts that control TAM function. Pharmacological inhibition of FTO has been reported to promote pro-inflammatory macrophage polarization and enhance the efficacy of immune checkpoint blockade therapies (107). Selective modulation of demethylase activity could shift TAMs from an M2-like immunosuppressive state toward an M1-like anti-tumor state, providing synergistic effects with existing immunotherapies (108).

### 5.3 Targeting m6A “readers” (binding proteins)

Readers such as YTHDF1, YTHDF2, and IGF2BP family proteins interpret m6A marks to regulate RNA stability and translation. Modulation of reader proteins in TAMs can alter the expression of key cytokines, chemokines, and immune checkpoint molecules. For example, interfering with YTHDF2 in TAMs may reduce the stability of transcripts promoting immunosuppression, thereby enhancing T cell-mediated anti-tumor responses. Targeting readers could be combined with other epigenetic or metabolic interventions to synergistically remodel TAM function (99).

### 5.4 Combination strategies and clinical translation

Beyond targeting individual m6A regulators, combination approaches offer additional therapeutic potential. Integrating m6A modulation with immune checkpoint inhibitors, adoptive T cell therapy, or conventional chemotherapy may overcome the immunosuppressive TME. Furthermore, nanoparticle-based delivery systems or TAM-specific targeting vectors could improve specificity and reduce off-target effects, facilitating translation into clinical applications.

In summary, m6A RNA modification represents a versatile target for reprogramming TAMs and enhancing anti-tumor immunity. A mechanistic understanding of how writers, erasers, and readers regulate TAM function provides a foundation for the rational design of m6A-based therapeutic strategies. Future studies should focus on the development of selective modulators, combination regimens, and TAM-targeted delivery platforms to fully realize the clinical potential of m6A-targeted therapy in cancer.

## 6 Challenges and future perspectives

Despite remarkable progress in recent years in understanding the role of m6A modification in immune cell biology, its functions and mechanisms in tumor-associated macrophages (TAMs) remain fraught with challenges. First, the high heterogeneity of TAMs constitutes a major barrier. TAMs arise from diverse origins, including monocyte-derived macrophages and tissue-resident macrophages, and display distinct functional states depending on tumor type and spatial niche. This heterogeneity makes it difficult to generalize the role of m6A modifications under a single paradigm, as their regulatory effects are more likely to be cell type– or even tumor-specific. Second, technological limitations need to be overcome. Conventional transcriptomics provides only bulk-level average signals, making it difficult to resolve m6A regulation at the single-cell level. With the rapid advances in single-cell transcriptomics and single-cell epitranscriptomics, future studies may achieve fine mapping of the spatial distribution and dynamic changes of m6A modifications. However, the sensitivity and resolution of current single-cell m6A detection remain limited. Integrating single-cell m6A profiling with spatial omics, proteomics, and other multi-omics approaches will be an important direction for advancing this field. From a translational perspective, combining m6A regulation with TAM-targeted therapies holds great promise. Drugs targeting m6A regulators (such as METTL3, FTO, and ALKBH5) are under development, while therapeutic strategies directed at TAMs—such as depletion, blockade of recruitment, or functional reprogramming—are already being tested in clinical settings. Therefore, the combination of m6A-targeting drugs with TAM-directed immunotherapies may represent a novel paradigm for future cancer treatment. Finally, future research should further elucidate the tumor-type–specific differences in the m6A–TAM axis. Given that the immune microenvironment varies substantially across cancer types, the role of m6A modification in TAM polarization, metabolic reprogramming, and immune regulation is likely to be context-dependent. Dissecting these differences will not only help uncover the general principles governing the m6A–TAM network but also provide a theoretical basis and novel targets for precision immunotherapy.

## 7 Conclusion

N6-methyladenosine (m6A), the most prevalent epitranscriptomic modification in eukaryotic RNA, has gained increasing attention for its role in tumor immune regulation. Accumulating evidence indicates that m6A modification profoundly influences the polarization state and functions of tumor-associated macrophages (TAMs) by regulating RNA stability, splicing, translation, and degradation at the post-transcriptional level (79, 89). Specifically, m6A modifications mediate the dynamic transition of TAMs between tumor-promoting (M2-like) and tumor-suppressive (M1-like) states, thereby playing pivotal roles in immunosuppression, tumor angiogenesis, metastasis, and response to immunotherapy. In summary, the m6A-TAM axis represents not only a critical entry point for understanding the plasticity of the tumor immune microenvironment but also a promising avenue for developing new therapeutic strategies and targets. With the ongoing advances in single-cell epitranscriptomics and the development of m6A-targeting drugs, precise modulation of TAM functions may become feasible, ushering in a new era of tumor immunotherapy.
